# Interaction With 14-3-3 Correlates With Inactivation of the RIG-I Signalosome by Herpesvirus Ubiquitin Deconjugases

**DOI:** 10.3389/fimmu.2020.00437

**Published:** 2020-03-12

**Authors:** Soham Gupta, Päivi Ylä-Anttila, Tatyana Sandalova, Adnane Achour, Maria G. Masucci

**Affiliations:** ^1^Department of Cell and Molecular Biology, Karolinska Institutet, Stockholm, Sweden; ^2^Division of Infectious Diseases, Department of Medicine Solna, Karolinska Institutet, Karolinska University Hospital, Stockholm, Sweden; ^3^Science for Life Laboratory, Stockholm, Sweden

**Keywords:** herpesvirus deubiquitinase, TRIM25 regulation, 14-3-3, type I interferon, RIG-1

## Abstract

The hijacking of cellular function through expression of proteins that interfere with the activity of cellular enzymes and regulatory complexes is a common strategy used by viruses to remodel the cell environment in favor of their own replication and spread. Here we report that the ubiquitin deconjugases encoded in the N-terminal domain of the large tegument proteins of Epstein-Barr virus (EBV), Kaposi Sarcoma herpesvirus (KSHV) and human cytomegalovirus (HCMV), but not herpes simplex virus-1 (HSV-1), target an early step of the IFN signaling cascade that involves the formation of a trimolecular complex with the ubiquitin ligase TRIM25 and the 14-3-3 molecular scaffold. Different from other homologs, the HSV-1 encoded enzyme fails to interact with 14-3-3, which correlates with failure to promote the autoubiquitination and sequestration of TRIM25 in cytoplasmic aggregates, and inability to block the activation and nuclear translocation of the IRF3 transcription factor. These findings highlight a key role for 14-3-3 molecular scaffolds in the regulation of innate immune response to herpesvirus infections and points to a possible target for the development of a new type of antivirals with applications in a broad spectrum of human diseases.

## Introduction

Post-translational modifications by covalent conjugation of ubiquitin and ubiquitin-like polypeptides play a key role in the regulation of a variety of cellular functions including the cellular and organismal response to infection (1). The recognition of pathogen-associated molecular patterns (PAMPS) by pattern recognition receptors (PRRs) initiates signaling cascades that are tightly regulated by ubiquitination and deubiquitination events (2). Following recognition of viral nucleic acids by members of the RIG-I-like receptor family, ubiquitination-dependent events mediates the transcriptional activation of type I interferon (IFN) that regulates the expression of a variety of IFN-stimulated genes (ISGs) whose products inhibit virus replication and decrease the cell susceptibility to infection (3). Given the central role of the IFN response in the control of infections, it is not surprising that viruses have evolved a variety of strategies for limiting IFN production. While the signaling events targeted by individual viruses are different, they often converge on preventing the phosphorylation, dimerization, and nuclear translocation of the transcription factors Interferon Response Factor (IRF)-3 and IRF7 (4, 5). Ubiquitination of the RIG-I family members by tripartite motif (TRIM) E3 ligases is a critical step in this signaling cascade that is often targeted by viruses. Failure to ubiquitinate RIG-I prevents its translocation to Mitochondrial Anti-Viral Signaling proteins (MAVS), blocking downstream events that, via recruitment of TNF-Receptor Associate Factor (TRAF)-3 and the TRAF family member-associated NF-kappa B activator (TANK), trigger the activation of TANK binding kinase-1 (TBK1), and I-kappa B kinase epsilon (IKK-epsilon) that phosphorylate IRF3 and IRF7 (6).

Previous studies implicate the cysteine proteases encoded in the N-terminal domain of the large tegument proteins of herpes viruses in the inhibition of type I IFN responses (7–15). In spite of very limited sequence homology, the enzymes encoded by Herpes simplex virus (HSV-UL36), Human cytomegalovirus (HCMV-UL48), Kaposi sarcoma virus (KSHV-ORF64), and Epstein-Barr virus (EBV-BPLF1) are functionally similar and exhibit potent ubiquitin- and NEDD8-specific deconjugase activities (16, 17). Members of this viral enzyme family were shown to interfere with the ubiquitination of various components of the IFN signaling pathway. Thus, KSHV-ORF64 and EBV-BPLF1 inhibit the ubiquitination of RIG-I (7, 12, 15), whereas TRAF3 ubiquitination is affected by HSV-UL36 and HCMV-UL48 (8, 14), suggesting that the viruses may have evolved different strategies to promote infection and replication in different cell types.

While attempting to elucidate the molecular interaction involved in the inhibition of the IFN response by the EBV-encoded BPLF1, we found that the catalytic domain of BPLF1 is recruited to a trimolecular complex that includes the TRIM25 ligase and members of the 14-3-3 family of molecular scaffolds (7). This promotes the activation and auto-ubiquitination of the ligase and the sequestration of TRIM25 in cytoplasmic aggregates, which correlates with failure to ubiquitinate RIG-I and functional inactivation of the IFN signaling pathway (18). We also found that binding of BPLF1 to 14-3-3 is essential for the formation of TRIM25 aggregates as well as for inhibition of the IFN response. The interaction site was mapped to acidic residues within BPLF1 helix-2 that are relatively well-conserved in the homologs encoded by other herpesviruses and may therefore provide an interesting target for the development of small molecule inhibitors (18).

In this investigation, we sought to elucidate whether the BPLF1 homologs encoded by HCMV, KSHV and HSV-1 share similar capacity to inhibit the IFN response by targeting the 14-3-3/TRIM25 complex. We found that the catalytic domains of HCMV-UL48 and KSHV-ORF64 resemble BPLF1 in their ability to induce the sequestration of TRIM25 in cytoplasmic aggregates that proxy for inhibition of the IFN response while this property was not shared by the catalytic domain of HSV-UL36. The failure of UL36 to induce TRIM25 autoubiquitination and the formation of aggregates is likely to be attributed to strongly reduced interaction with 14-3-3, probably due to critical differences in solvent exposed residues within helix-2.

## Methods

### Chemicals

DL-Dithiothreitol (DTT, D0632), N-Ethylmaleimide (NEM, E1271), Iodoacetamide (I1149), IGEPAL CA-630 (NP40, I3021), Triton X-100 (T9284), bovine serum albumin (BSA, A7906), Sodium dodecyl sulfate (SDS, L3771), Tween-20 (P9416), Ethylenediaminetetraacetic acid disodium salt dehydrate (EDTA, E4884), and Trizma base (Tris, 93349) were purchased from Sigma-Aldrich (St. Louis, MO, USA). Complete protease inhibitor cocktail (04693116001) and phosphatase inhibitor cocktail (04906837001) were purchased from Roche Diagnostic (Mannheim, Germany). Ciprofloxacin (17850) was purchased from Fluka (Buchs, Switzerland).

### Antibodies

Antibodies and their manufacturers were: mouse anti-FLAG clone M2 (1:5000, IF: 1:500; F1804) from Sigma-Aldrich; mouse anti-HA clone 12CA5 (1:2000; 11583816001) from Roche; mouse anti-pan 14-3-3 clone H-8 (1:1000; sc-1657), from Santa-Cruz Biotechnology (Santa Cruz, CA, USA); rabbit anti-IRF-3 clone D6I4C (1:1000, IF 1:200; #11904) and mouse anti-GST clone 26H1 (1:1000, IF 1:100 #2624) from Cell-Signaling Technologies (Danvers, MA, USA); rabbit anti TRIM25 clone EPR7315 (1:2000, IF: 1:100; ab167154) from Abcam (Cambridge, MA, USA); mouse anti-HA.11 clone 16B12 (1:1000; 901501) from BioLegend (San Diego, CA, USA). Alexa Fluor 488, 555, and 647 conjugated secondary antibodies were from Thermo Fisher (A21206, A31570, and A21447, respectively).

### Plasmids

Plasmids encoding 3xFLAG-BPLF1 (amino acid residues 1–235), the catalytic mutant BPLF1-C61A, and 3xFLAG-KSHV-ORF64 were described previously (16, 19). The plasmid expressing 3xFLAG-tagged HSV-UL36 (amino acid residues 1–293) was kindly provided by Lars Dolken, University of Wurzburg, Germany and the plasmid expressing 3xFLAG-HCMV-UL48 was kindly provided by Luka Cicin-Sain, Helmholtz Center for Infection Research, Braunschweig, Germany (7). The plasmid encoding for GST-2CARD was kindly provided by Jae Jung, University of Southern California, USA and the plasmid pcDNA3.0-HA-TRIM25 encoding the full length human TRIM25 gene was a gift from Dong-Er Zhang (Addgene plasmid # 12452) (20).

### Cell Lines and Transfection

HeLa cells (ATCC RR-B51S) were cultured in Dulbecco's minimal essential medium (DMEM, Sigma-Aldrich), supplemented with 10% FCS (Gibco-Invitrogen), ciprofloxacin (10 μg/ml) and maintained in a 37°C incubator in 5% CO_2_. Plasmid transfection was performed using the jetPEI DNA transfection reagent (Polyplus transfection; Illkirch, France) as recommended by the manufacturer.

### Immunoblotting and Immunoprecipitation

For immunoblotting and co-immunoprecipitation, cells harvested 48 h post transfection were lysed in NP-40 lysis buffer (50 mM Tris-HCl pH 7.6, 150 mM NaCl, 5 mM MgCl2, 1 mM EDTA, 1 mM DTT, 1% Igepal, 10% glycerol) supplemented with protease inhibitor cocktail 20 mM NEM and 20 mM Iodoacetamide and phosphatase inhibitor cocktail whenever required. Protein concentration was measured with a protein assay kit (Bio-Rad Laboratories). For co-immunoprecipitation of Flag-tagged herpesvirus DUB homologs, the cell lysates were incubated for 4 h with anti-FLAG agarose affinity gel (A-2220; Sigma), followed by washing with lysis buffer and elution with 3x-FLAG peptide (F4799; Sigma) at a concentration of 300 μg/ml. RIG-I-2CARD ubiquitination was determined by immunoprecipitating ectopically expressed GST-2CARD using Glutathione Sepharose 4B beads (Amersham Biosciences) under denaturing conditions. To resolve protein complexes, cell pellets were lysed in 100 μl NP-40 lysis buffer (50 mM Tris-HCl pH 7.6, 150 mM NaCl, 1 mM EDTA, 1 mM DTT, 1% Igepal) supplemented with 1% sodium dodecyl sulfate (SDS). Before immunoprecipitation NP-40 buffer was added to reach a final concentration of 0.1% SDS. Immunocomplexes were washed with lysis buffer containing 0.1% SDS. Elution was performed by boiling with 2x SDS-PAGE loading buffer. Equal amounts of proteins were fractionated in polyacrylamide Bis-Tris 4–12% gradient gels (Invitrogen). After transfer to poly-vinylidene difluoride (PVDF) membranes (Millipore), the blots were blocked in Tris-buffered saline containing 5% non-fat skimmed milk powder and 0.1% Tween 20 and incubated with primary antibodies for either 1 h at room temperature or overnight at 4°C, followed by incubation for 1 h with the appropriate horseradish peroxidase-conjugated secondary antibodies. The complexes were visualized by chemiluminescence (ECL; GE Healthcare).

### Immunofluorescence and Confocal Microscopy

HeLa cells were grown to semi-confluency in Dulbecco's minimal essential medium containing 10% fetal calf serum and 100 μg/ml ciprofloxacin on glass cover slips and transfected with the indicated plasmids using the jetPEI kit as recommended by the manufacturer. After 24 h the cells were fixed in 4% paraformaldehyde (Merck, 100496). To stain endogenous TRIM25, the cells were permeabilized with 0.05% Triton X-100 in PBS for 5 min at room temperature (RT), blocked with 3% BSA in PBST (0.05% Triton X-100 in PBS) for 30 min, and labeled with rabbit anti-TRIM25 and mouse anti-FLAG antibodies diluted in blocking buffer. To stain endogenous IRF3 in GST-2CARD activated cells, cells were permeabilized using 0.1% Triton X-100 in PBS, followed by blocking with 0.12% glycine (Fisher Scientific, G46-1) in PBS for 10 min, and 3% bovine serum albumin (BSA, Sigma, A7906) in PBS for 15 min at room temperature. The cells were triple labeled in 3% BSA-PBS using rabbit anti-IRF3, mouse anti-GST and goat anti-FLAG antibodies and then incubated with appropriate Alexa Fluor 488, 555, or 647 conjugated secondary antibodies. The coverslips were mounted cell side down on object glasses with Mowiol (Calbiochem, 475904) containing 50 mg/ml 1,4-diazabicyclo [2.2.2]octane (Dabco; Sigma, D-2522) as anti-fading agent and 2 ug/ml Hoechst 33258 (Sigma, 861405) or DAPI to stain the nuclei (not shown). The samples were imaged using a confocal scanning laser microscope (Zeiss LSM800 META) and 2.5 μm optical sections were acquired.

### Molecular Modeling

Structural models of the DUB module of BPLF1 and the homologs encoded by HCMV, HSV, and KSHV were made on the template of the crystal structure of the DUB module of murine cytomegalovirus (MCMV) M48 PBD code 2J7Q (21), using the homology modeling option of SwissModel (https://www.swissmodel.expasy.org/) (22). Docking of BPLF1on the 14-3-3 dimer was performed using the ClusPro docking server [https://cluspro.bu.edu/login.php, (23). The dimer found in the crystal structure of 14-3-3 in the open conformation PDB code 2C23, Yang et al. (25)] and the coordinates of the homology model of BPLF1 were used in docking experiments. The distance between residues 14-3-3 Val181 and BPLF1 Glu90 was restrained to the range 3–7Å during docking.

## Results

### Induction of TRIM25 Auto-Ubiquitination and the Formation of Aggregates

**Figure 1:**
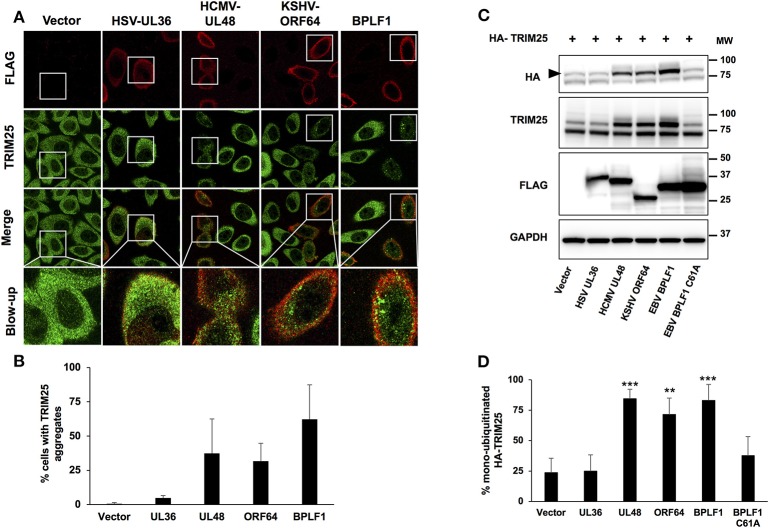
TRIM25 aggregate formation and autoubiquitination in cells expressing the viral ubiquitin deconjugases. HeLa cells were transfected with FLAG-tagged versions of the viral enzymes and cells were harvested 24 h after transfection. **(A)** Representative micrographs illustrating the formation of TRIM25 aggregates. Confocal images were obtained with a 63 x lens objective magnification. TRIM25 is green, BPLF1 is red. **(B)** Quantification of the number of FLAG expressing cells exhibiting TRIM25 aggregates. The mean ± SD of two independent experiments is shown. **(C)** Representative western blots illustrating the induction of TRIM25 autoubiquitination in cells expressing the viral enzymes. Hela cells were co-transfected with HA-TRIM25 plasmid and plasmids encoding the indicated catalytically active FLAG-tagged N-terminal domains of herpesvirus deconjugases along with catalytically inactive FLAG-BPLF1-C61A. Western blots were probed with the HA antibody. A band shift corresponding to monoubiquitinated TRIM25 (indicated by a black arrow) was detected in cells transfected with BPLF1, UL48, and ORF64 but not in cells transfected with HSV-UL36. The western blots from one representative experiment out of 4 are shown. **(D)** The intensity of the TRIM25 and the mono-ubiquitinated TRIM25 was quantified by densitometry and the percentage of mono-ubiquitinated TRIM25 was calculated. The means ± SD of 4 experiments are shown. Statistical analysis was performed using the Student's *t*-test: **P ≤ 0.01 and ***P ≤ 0.001.

We have previously reported that the catalytic domain of the EBV large tegument protein BPLF1 inhibits IFN signaling by inducing the formation of a trimolecular complex including 14-3-3 and the ubiquitin ligase TRIM25, which correlates with failure to ubiquitinate RIG-I and functional inactivation of the RIG-I signalosome (7, 18). This effect is critically associated with the induction of TRIM25 autoubiquitination and formation of TRIM25 aggregates that are distinct from the stress granules induced by viral infection and may serve as proxy for the inhibitory effect of the viral enzyme (18). In order to investigate whether this property is shared by the homologs encoded by other herpesviruses, HeLa cells were transfected with the FLAG-tagged versions of the catalytic domains of EBV-BPLF1, HSV-UL36, HCMV-UL48, and KSHV-ORF64 and the formation of endogenous TRIM25 aggregates was monitored 24 h after transfection by staining with antibodies specific for TRIM25. Of note, the four homologs showed comparable catalytic activity as confirmed by labeling with ubiquitin specific functional probes (Figure S1) As illustrated by the representative micrographs shown in Figure 1A, and quantification of the % positive cells in two independent experiments shown in Figure 1B, small TRIM25 aggregates were readily detected throughout the cytoplasm of cells expressing EBV-BPLF1, KSHV-ORF64, and HCMV-UL48 while diffuse cytoplasmic fluorescence was consistently observed in cells expressing HSV-UL36.

We have demonstrated that the formation of TRIM25 aggregates is critically dependent on the capacity of BPLF1 to induce TRIM25 auto-ubiquitination and promote the accumulation of mono/di-ubiquitinated species derived from the trimming of K48-linked polyubiquitin chains (18). In order to assess the validity of this observation in cells expressing the BPLF1 homologs, HeLa cells were co-transfected with HA-tagged TRIM25 and FLAG-tagged EBV-BPLF1, HSV-UL36, HCMV-UL48, and KSHV-ORF64. Western blots of cells harvested 48 h after transfection were probed with antibodies specific for TRIM25 and the HA-tag (Figures 1C,D). In line with previous reports (12), a weak band corresponding to mono-ubiquitinated TRIM25 was detected in cells expressing the HA-TRIM25 construct, probably due to auto-activation of the overexpressed ligase. As expected, the intensity of the mono-ubiquitinated TRIM25 band was significantly increased in cells expressing BPLF1 but not the catalytically inactive BPLF1-C61A mutant. The amount of mono-ubiquitinated TRIM25 was also strongly increased in cells expressing KSHV-ORF64 and HCMV-UL48 resulting in more than 70% mono-ubiquitinated TRIM25 (Figures 1C,D). In contrast, cells expressing HSV-UL36 showed levels of TRIM25 mono-ubiquitination comparable to those detected in cells transfected with empty vector or BPLF1-C61A mutant. Collectively, these findings confirm the association between the accumulation of mono-ubiquitinated TRIM25 and the formation of aggregates and highlight the different functional behavior of the catalytic domain of HSV-UL36.

### Inhibition of IFN Signaling

**Figure 2:**
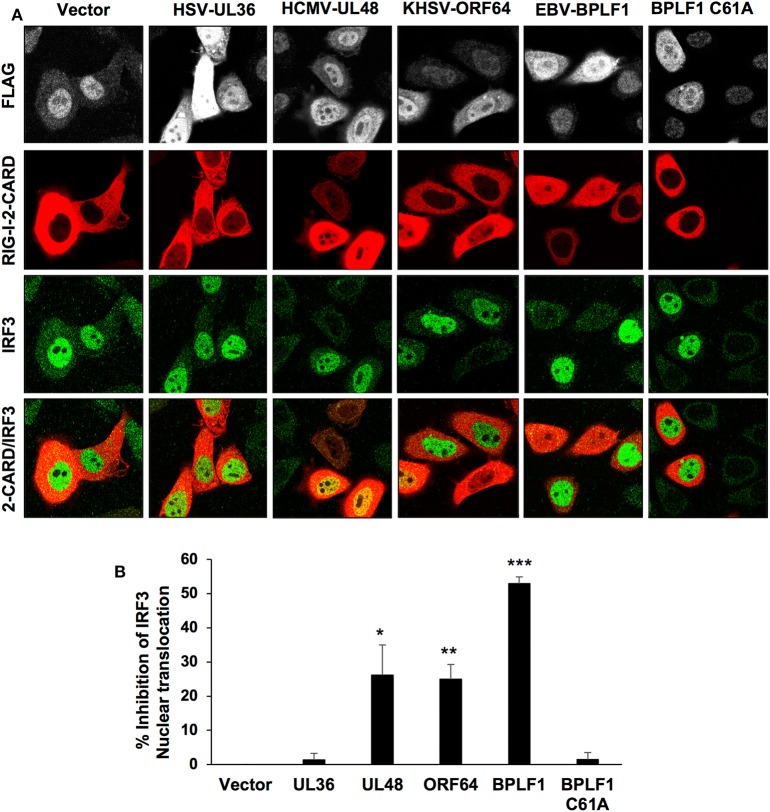
Inhibition of the type I IFN response in cells expressing the viral enzymes. The IFN response was triggered in HeLa cells transfected with the FLAG-tagged viral enzymes by co-transfection with a RIG-I-2CARD expressing plasmid and activation of the response was assessed by monitoring the nuclear translocation of the IRF3 transcription factor. **(A)** the cells were harvested 48 h after transfection and co-stained with antibodies against the FLAG-tag (gray) RIG-I-2CARD (red) and IRF3 (green). Confocal images were obtained with 63 x lens objective magnification. **(B)** Inhibition of IRF3 nuclear translocation in RIG-I-2CARD positive cells expressing the viral enzymes was calculated relative to the percentage of vector transfected nuclear IRF3 positive cells expressing RIG-I-2CARD. The mean ± SD of two independent experiments is shown. Statistical analysis was performed using the Student's *t*-test: *P ≤ 0.05, **P ≤ 0.01, and ***P ≤ 0.001.

Since the catalytic domain of HSV-UL36 failed to induce TRIM25 mono-ubiquitination and the formation of TRIM25 aggregates, we further investigated its capacity to inhibit the type I IFN response as assessed by activation and nuclear translocation of the IRF3 transcription factor. To this end, the interferon response was triggered by co-transfection of constitutively active RIG-I-2CARD in cells transfected with the catalytic domains of EBV-BPLF1, HSV-UL36, HCMV-UL48, or KSHV-ORF64. As illustrated by the representative micrographs shown in Figure 2A and quantification of two independent experiments (Figure 2B), IRF3 nuclear translocation was readily detected in virtually all vector or BPLF1-C61A transfected cells expressing RIG-1-2CARD while more than 50% inhibition of IRF3 translocation was observed in cells expressing catalytically active BPLF1. Significant levels of inhibition were also detected in cells expressing KSHV-ORF64 or HCMV-UL48 whereas there was virtually no inhibition in cells expressing HSV-UL36. Concordant results were obtained when the inactivation of the pathway was assessed by inhibition of RIG-I ubiquitination, with the strongest inhibition achieved in BPLF1-expressing cells and weakest inhibition in cells expressing the BPLF1-C61A catalytic mutant and HSV-UL36 (Figure S2). However, quantitative comparison is more uncertain in this type of assays due to possible artifacts of overexpression.

### The Surface Charge of Helix-2 Is Critical for Binding to 14-3-3 and Inhibition of IFN Signaling

**Figure 3:**
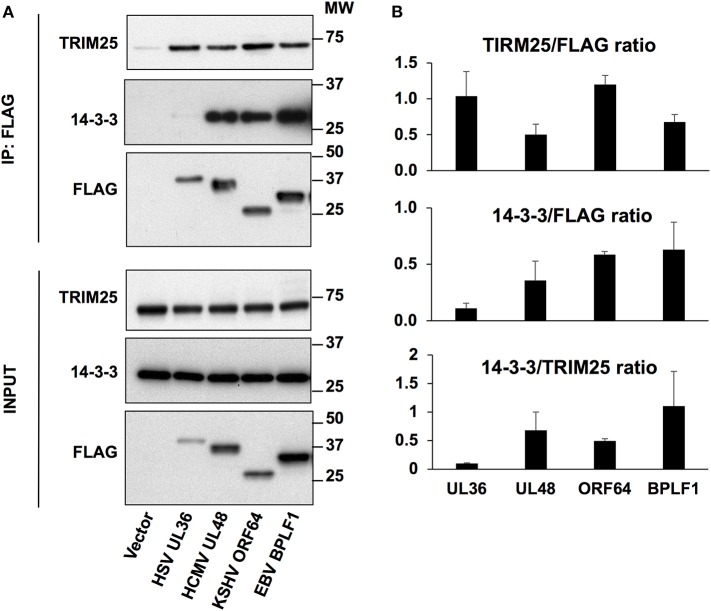
Interaction of the viral deconjugases with 14-3-3 and TRIM25. **(A)** HeLa cells were transfected with the indicated FLAG-tagged plasmids and cell lysates were immunoprecipitated with anti-FLAG conjugated agarose beads (IP). Western blots of the immunoprecipitates were probed with PAN 14-3-3, TRIM25 and FLAG specific antibodies. One representative experiment out of three is shown in the figure. **(B)** The intensity of the TRIM25, 14-3-3, and FLAG specific bands was quantified by densitometry and relative binding was calculated as the ratio between the intensity of the 14-3-3 or TRIM25 specific bands relative to FLAG. The lower panel represents the ratio between these two. The means ± standard error of three experiments are shown.

*In vitro* binding assays using purified proteins showed that EBV-BPLF1 independently interacts with both 14-3-3 and TRIM25 (18). However, an EBV-BPLF1 mutant that fails to bind to 14-3-3 also lost the capacity to induce TRIM25 autoubiquitination and the formation of aggregates (18), pointing to 14-3-3 as an essential co-factor for inhibition of this step of the IFN response. We asked therefore whether the BPLF1 homologs share the capacity to interact with 14-3-3 and TRIM25. To this end, HeLa cells were transfected with Flag-tagged versions of the N-terminal catalytic domains of EBV-BPLF1, HSV-UL36, HCMV-UL48, and KSHV-ORF64 and FLAG immunoprecipitates were probed with antibodies specific for 14-3-3 and TRIM25. As illustrated by the representative western blots shown in Figure 3A, and densitometry quantification of the specific bands in three independent experiments (Figure 3B), all the homologs interacted with comparable efficiency with TRIM25 while HSV-UL36 showed a remarkably weaker interaction with 14-3-3. Thus, the ratio between the FLAG co-immunoprecipitated 14-3-3 and TRIM25 measured by the intensity of the specific bands was significantly lower in HSV-UL36 compared to the remaining homologs (Figure 3B). When considering all the homologs, the 14-3-3/TRIM25 ratio showed a strong positive correlation with the levels of TRIM25 mono-ubiquitination (*r* = 0.94), the formation of TRIM25 aggregates (*r* = 0.97) and inhibition of IRF3 nuclear translocation (*r* = 0.93) (Figure S3). Collectively, these results support our previous findings and confirm the pivotal role of 14-3-3 interaction in the regulation of TRIM25 autoubiquitination, formation of TRIM25 aggregates and inhibition of IFN signaling by the viral ubiquitin deconjugases.

**Figure 4:**
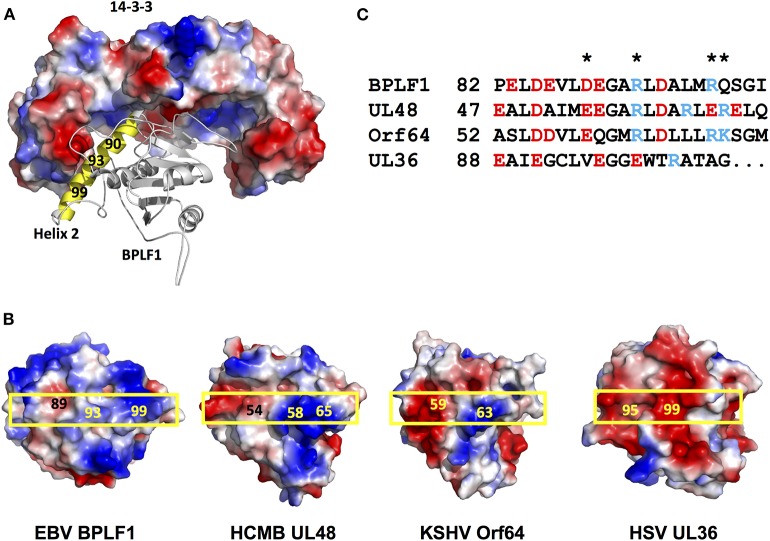
Distribution of the charged residues on helix 2 of the viral deconjugases. **(A)** The Cluspro top hit three-dimensional molecular model of the binary complex of the 14-3-3 dimer (shown as surface colored by electrostatic potential: positive charge, blue; negative charge, red, non-charged, white), and BPLF1 (cartoon) illustrates how solvent exposed residues of BPLF1 helix 2 (yellow) may participate in complex formation. **(B)** The electrostatic surface potential of the molecular models of the N-terminal domains of the viral deconjugases reveal striking differences between UL36 and the other proteins. The position of helix 2 on the surface of each deconjugase is indicated by a yellow box. **(C)** Alignment of the helix 2 residues for the four different viruses. The charged solvent accessible residues indicated in 4B are highlighted (*).

14-3-3 proteins serve as molecular scaffolds by forming homo/heterodimers that establish electrostatic interactions with charged residues on client proteins, which may stabilize protein conformations (24) or promote the formation of protein complexes (25). To this end, the 14-3-3 substrate binding groove comprises a positively charged “corner” where phosphorylated or acidic residues can bind, and “claws” where negatively charged residues are alternated with non-charged residues, providing ligand specificity [Yaffe et al. (24); Yang et al. (25)]. Based on mutation analysis, molecular modeling and *in silico* docking experiments we have earlier suggested that 14-3-3 could stabilize the interaction of BPLF1 with TRIM25 via the interaction of the adjacent binding sites in the substrate binding groove of the 14-3-3 dimer with acidic motifs located at the tip of the TRIM25 coiled-coil domain and helix-2 of BPLF1, respectively (18). As illustrated by a tri-dimensional model of the binary complex of 14-3-3 with the N-terminal domain of BPLF1 shown in Figure 4A, the acidic N-terminus of helix-2 is positioned close to the phospho-binding site in 14-3-3, while the positively charged middle part of helix-2 could be positioned close to the negatively charged “claws.”

Although showing <30% sequence homology, the N-terminal domains of the herpesvirus large tegument proteins exhibit very similar fold resulting in the highly conserved C-box and H-box domains forming the catalytic groove of the enzyme, with the relatively well-conserved helix-2 pointing in the opposite direction (18, 19). Having shown that helix-2 mediates the interaction of BPLF1 with 14-3-3 we asked whether variations in this domain could explain the different behavior of the homologs. Molecular models of the N-terminal domains of EBV-BPLF1, HSV-UL36, HCMV-UL48, and KSHV-ORF64 created on the template of the crystal structure of the murine cytomegalovirus M48 homolog [Schlieker et al. (21)] are shown in Figure 4B and the alignment of residues forming helix-2 is presented in Figure 4C. The models revealed significant differences in the length and charge distribution of the solvent exposed surface of helix-2 (Figure 4B boxed region). In particular, while the helix-2 of BPLF1, UL48, and ORF64 display similar negatively charged N-terminus and positively charged C-terminus, in UL36 helix-2 is one helical turn shorter and has evenly distributed negative charges all over the entire length of helix-2, which could preclude interaction with the negatively charged “claw” of 14-3-3.

## Discussion

Previous studies have highlighted a novel strategy by which BPLF1, the ubiquitin deconjugase encoded in the N-terminal domain of EBV large tegument protein, inhibits an early step of the type I IFN response by targeting the interaction of the TRIM25 ubiquitin ligase with the 14-3-3 molecular scaffold. Here we report that the functional homologs encoded by HCMV and KSHV share the same capacity to target the 14-3-3/TRIM25 complex, whereas the HSV-1 encoded homolog interacts poorly with 14-3-3, which results in failure to inactivate TRIM25-mediated signaling.

The 14-3-3 molecular scaffolds regulate the IFN response by stabilizing the interaction of activated RIG-I with the TRIM25 ubiquitin ligase, which facilitates RIG-I ubiquitination and translocation to MAVS for downstream signaling (26, 27). The recruitment of BPLF1 to the 14-3-3/TRIM25 complex promotes the autoubiquitination and sequestration of the ligase into aggregates whose appearance correlates with inhibition of RIG-I ubiquitination and interruption of the signaling cascade that leads to activation and nuclear translocation of the IRF3 transcription factor (18). Recently, we could map the BPLF1 domain involved in this interaction to the solvent exposed negatively charged Glu residues in position 86 and 90 of helix-2 whose mutation to Arg does not affect the catalytic function but abolishes the capacity of the viral enzyme to inhibit the IFN response (18). These residues were previously shown to play an important role in the interaction of BPLF1 with cullin ligases (19), which is critical for the capacity of the viral protein to regulate virus replication and the release of infectious virus particles. The BPLF1-D86-90R mutations did not affect the interaction of BPLF1 with TRIM25 but severely impaired binding to 14-3-3, pointing to 14-3-3 as an essential co-factor for inhibition of the IFN response via functional inactivation of TRIM25.

The results of this investigation support this conclusion. We have found that while the N-terminal catalytic domains of the BPLF1 homologs encoded by HCMV and KSHV share the capacity of BPLF1 to promote the autoubiquitination and sequestration of TRIM25 into aggregates, and to inhibit RIG-I ubiquitination and IRF3 nuclear translocation, the homolog encoded by HSV-1 did not exhibit these properties. In line with the notion that interaction with 14-3-3 is a key requirement for the effect of the viral enzymes on TRIM25 function and subcellular localization, HSV-UL36 resembled the BPLF1-D86-90R mutant in showing efficient interaction with TRIM25 but strongly impaired binding to 14-3-3. It is noteworthy that although helix-2 is the only region beside the catalytic C- and H-boxes where the N-terminal domains of the herpesvirus large tegument proteins show some sequence similarity, both HSV-1 and HSV-2 diverge significantly from the consensus (18, 19). In particular, HSV-1 helix-2 is one helical turn shorter compared to the other homologs and does not exhibit the negatively charged N-terminus and positively charged C-terminus that, based on molecular docking, mediate the interaction with the substrate binding groove of 14-3-3. The importance of alternating positive and negatively charged patches is substantiated by the observation that binding to 14-3-3 is abolished by substitution of the negatively charged Glu residues in the N-terminus of BPLF1 helix2 positively charged Arg (18). Attempts to experimentally validate the model by generating 14-3-3 binding variant of HSV-UL36 by selected amino acid substitutions or by swap of the entire helix2 have been so far unsuccessful, probably due to failure to preserve of precise positioning of helix2 required for correct molecular folding and catalytic activity.

The difference between the HSV-1 and the other herpesvirus homologs is somewhat surprising since inhibition of the type I IFN response is a key requirement for herpes virus persistence and pathogenesis (28–31). It is important to note that, while BPLF1 and the homologs are huge proteins of more than 3,000 amino acids (21, 32), our experiments were performed with constructs expressing N-terminal catalytic domains of ~300 amino acids. We have previously shown that the full length BPLF1 is processed by caspase-1 during productive EBV infection giving rise to a catalytically active fragment of size corresponding to the construct used in our experiments (33). It is unclear whether the homologs undergo similar processing and, although unlikely, we cannot formally exclude the possibility that UL36 sequences that are not included in our construct may interact with the 14-3-3 or other sites in the 14-3-3/TRIM25 complex. It seems more likely that this herpesvirus homolog may target a different step of the signaling cascade as also indicated by the finding that UL36 deubiquitinates TRAF3, which inhibits the recruitment of the TBK1 kinase and subsequent activation of IRF3 and IRF7 (14). This signaling step is also affected by HCMV-UL48 along with the STING and TRAF6 mediated IFN response (8), suggesting that the viral deconjugases may act at multiple levels of the signaling cascade, possibly reflecting the need to adapt to different susceptible targets and types of infection. Of note, UL36 may also inhibit the IFN response independently of its deubiquitinase activity by targeting the IFN receptors IFNAR2 (34).

In conclusion, our study identifies the early step of the IFN signaling cascade exemplified by the interaction of 14-3-3 with the TRIM25 ligase as a common target for the inhibitory activity of EBV, KSHV and HCMV. This sheds light on the significant impact of these viral proteins on the virus lifecycle, including the establishment of persistent infection. Our work points toward 14-3-3 as a key player in the functional inactivation of TRIM25 in cells infected by β- and γ-herpesviruses. In view of the shared mechanism of action, interference with the recruitment of the viral enzymes to the 14-3-3/TRIM25 complex could provide a new strategy for potentiating the host innate immune response during the early phases of infection and virus reactivation.

## Data Availability Statement

The datasets generated for this study are available on request to the corresponding author.

## Author Contributions

SG designed the experiments, performed experimental work, analyzed the results, and wrote the first draft of the manuscript. PY-A designed and performed experimental work. TS built the structures and generated molecular models. AA contributed expertise in structural biology and molecular modeling. MM designed the study, supervised the experimental work, analyzed the results, and wrote the manuscript together with all co-authors.

### Conflict of Interest

The authors declare that the research was conducted in the absence of any commercial or financial relationships that could be construed as a potential conflict of interest.

## Supplementary Material

Figure S1 The viral enzymes are catalytically active. NP-40 lysates of cells expressing FLAG-tagged versions of the N-terminal domain of the of HSV-UL36, HCMV-UL48, KSHV-ORF64, and EBV-BPLF1 and its catalytic mutants were incubated for 1 h at 37°C with 0.5 μg of the Ub-VS functional probe. After fractionation by SDS-PAGE and blotting on PVDF membranes the viral proteins were detected with an anti-FLAG antibody. Enzymatic activity is confirmed by the appearance of a slower migrating species of size corresponding to cross-linked Ub-VS (indicated by a triangular pointer).

Figure S2 Ubiquitination of RIG-I-2CARD in cells expressing the viral deconjugases. **(A)** HeLa cells were co-transfected with the indicated FLAG-tagged herpesvirus deconjugases, constitutively active RIG-I-2CARD and HA-tagged ubiquitin where all Lys residues except K63 were mutated to Arg. RIG-I-2CARD was immunoprecipitated 48 h after transfection and ubiquitination was assessed by probing the immunoblots with an anti-HA antibody. One representative experiment out of three is shown. **(B)** The intensity of the ubiquitin blots was quantified by densitometry and the levels of ubiquitination in the presence of the viral enzyme were calculated relative to the empty vector. The mean ± SD of three experiments is shown.

Figure S3 Correlation between the interaction with 14-3-3 and TRIM25 and inhibition of the IFN response. Graphic representation of the relationship between the ratio of 14-3-3/TRIM25 co-immunoprecipitation (blue dotted line) and: TRIM25 mono-ubiquitination (gray line), the formation of TRIM25 aggregates (orange line), inhibition of IRF3 nuclear translocation (yellow line). The data are expressed in arbitrary units. Higher 14-3-3/TRIM25 ratio correlates with increased TRIM25 aggregate formation (*r* = 0.97), TRIM25 ubiquitination (*r* = 0.93) and inhibition of IRF3 nuclear translocation (*r* = 0.94).
