# Supplementary figures and images for: Interaction With 14-3-3 Correlates With Inactivation of the RIG-I Signalosome by Herpesvirus Ubiquitin Deconjugases
Source: Front Immunol. 2020 Mar 12;11:437. doi: 10.3389/fimmu.2020.00437 (PMC7080818; doi:10.3389/fimmu.2020.00437)

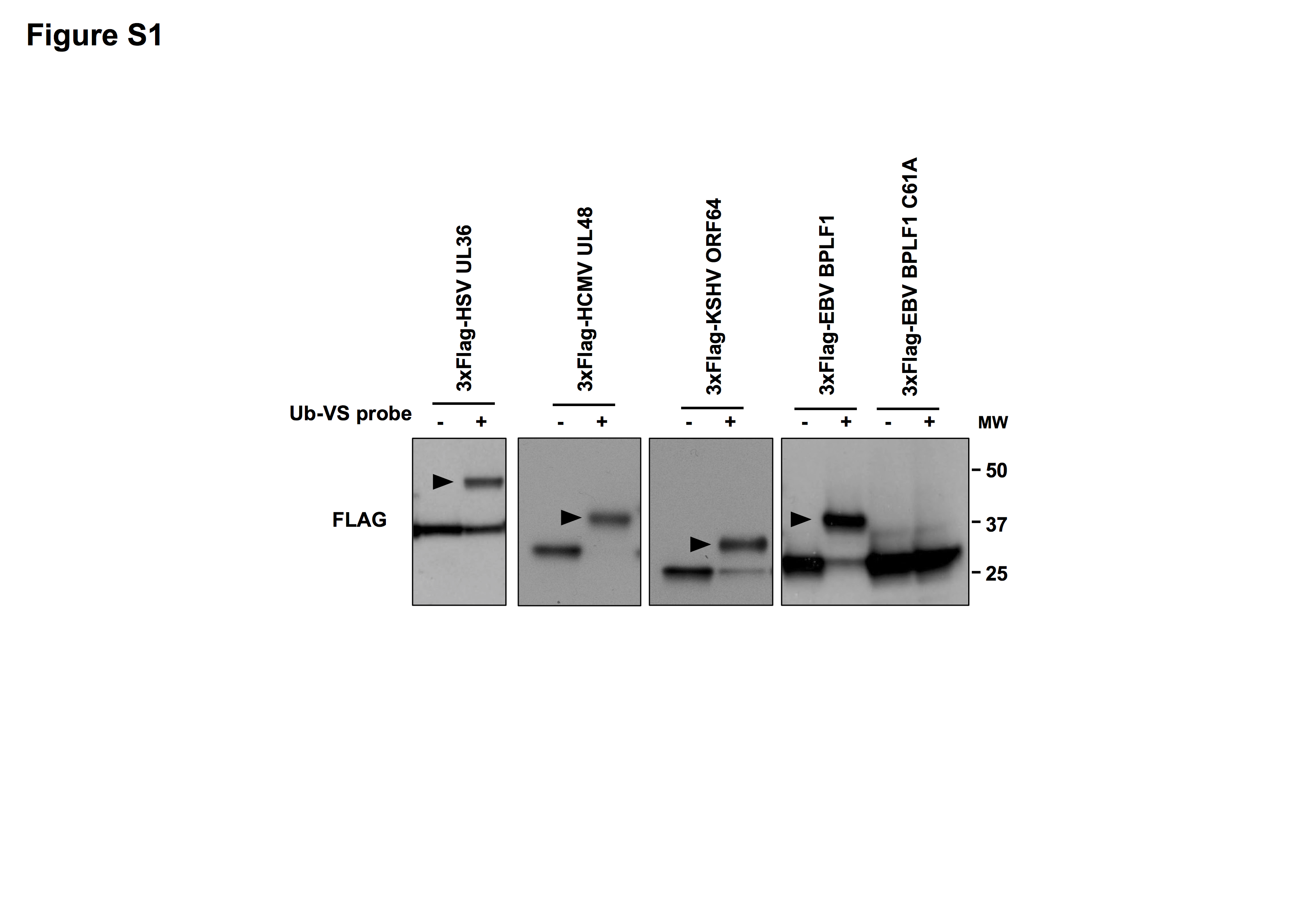

Supplement: Figure S1 — The viral enzymes are catalytically active. NP-40 lysates of cells expressing FLAG-tagged versions of the N-terminal domain of the of HSV-UL36, HCMV-UL48, KSHV-ORF64, and EBV-BPLF1 and its catalytic mutants were incubated for 1 h at 37°C with 0.5 μg of the Ub-VS functional probe. After fractionation by SDS-PAGE and blotting on PVDF membranes the viral proteins were detected with an anti-FLAG antibody. Enzymatic activity is confirmed by the appearance of a slower migrating species of size corresponding to cross-linked Ub-VS (indicated by a triangular pointer). [file Image_1.TIFF]

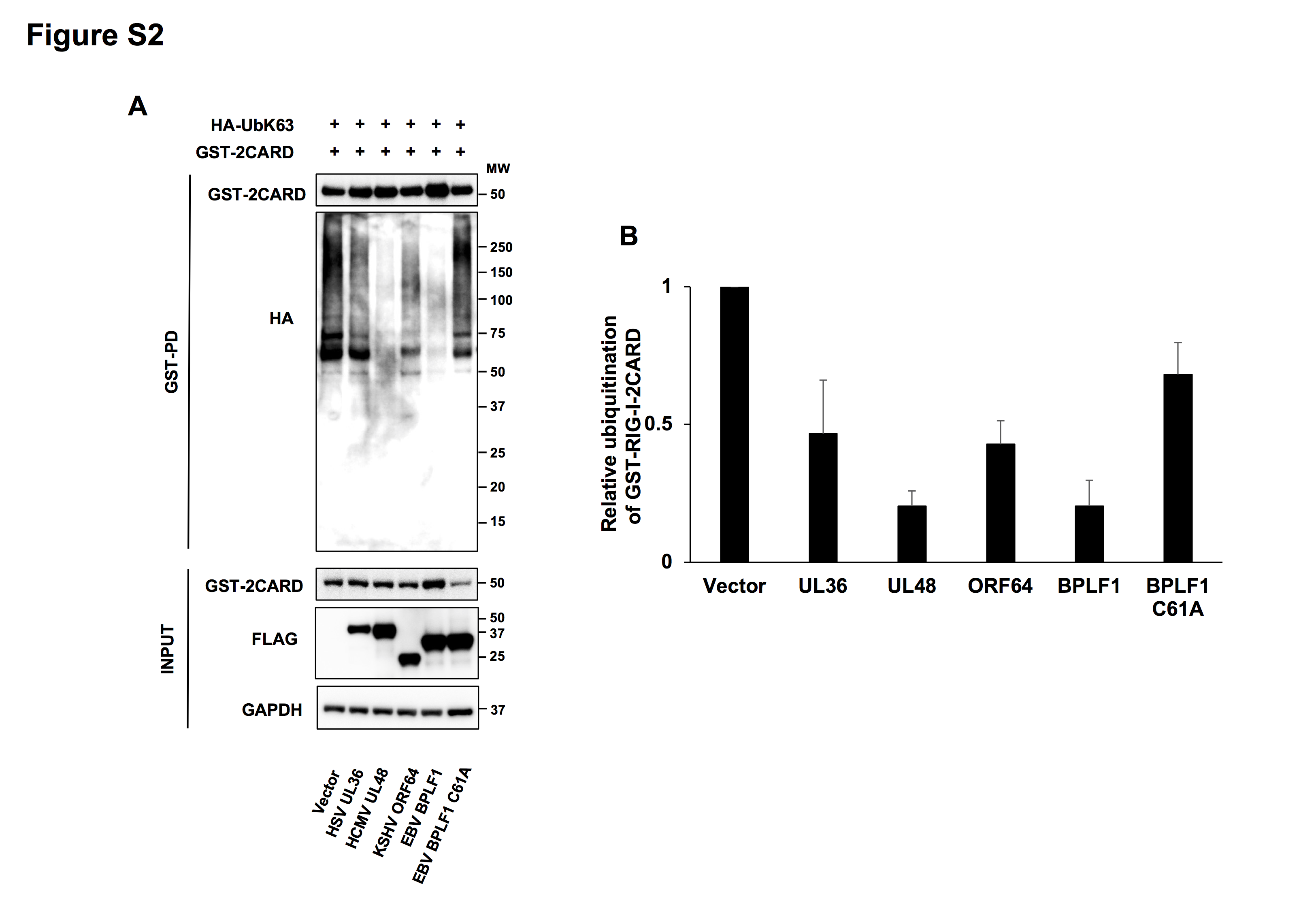

Supplement: Figure S2 — Ubiquitination of RIG-I-2CARD in cells expressing the viral deconjugases. (A) HeLa cells were co-transfected with the indicated FLAG-tagged herpesvirus deconjugases, constitutively active RIG-I-2CARD and HA-tagged ubiquitin where all Lys residues except K63 were mutated to Arg. RIG-I-2CARD was immunoprecipitated 48 h after transfection and ubiquitination was assessed by probing the immunoblots with an anti-HA antibody. One representative experiment out of three is shown. (B) The intensity of the ubiquitin blots was quantified by densitometry and the levels of ubiquitination in the presence of the viral enzyme were calculated relative to the empty vector. The mean ± SD of three experiments is shown. [file Image_2.TIFF]

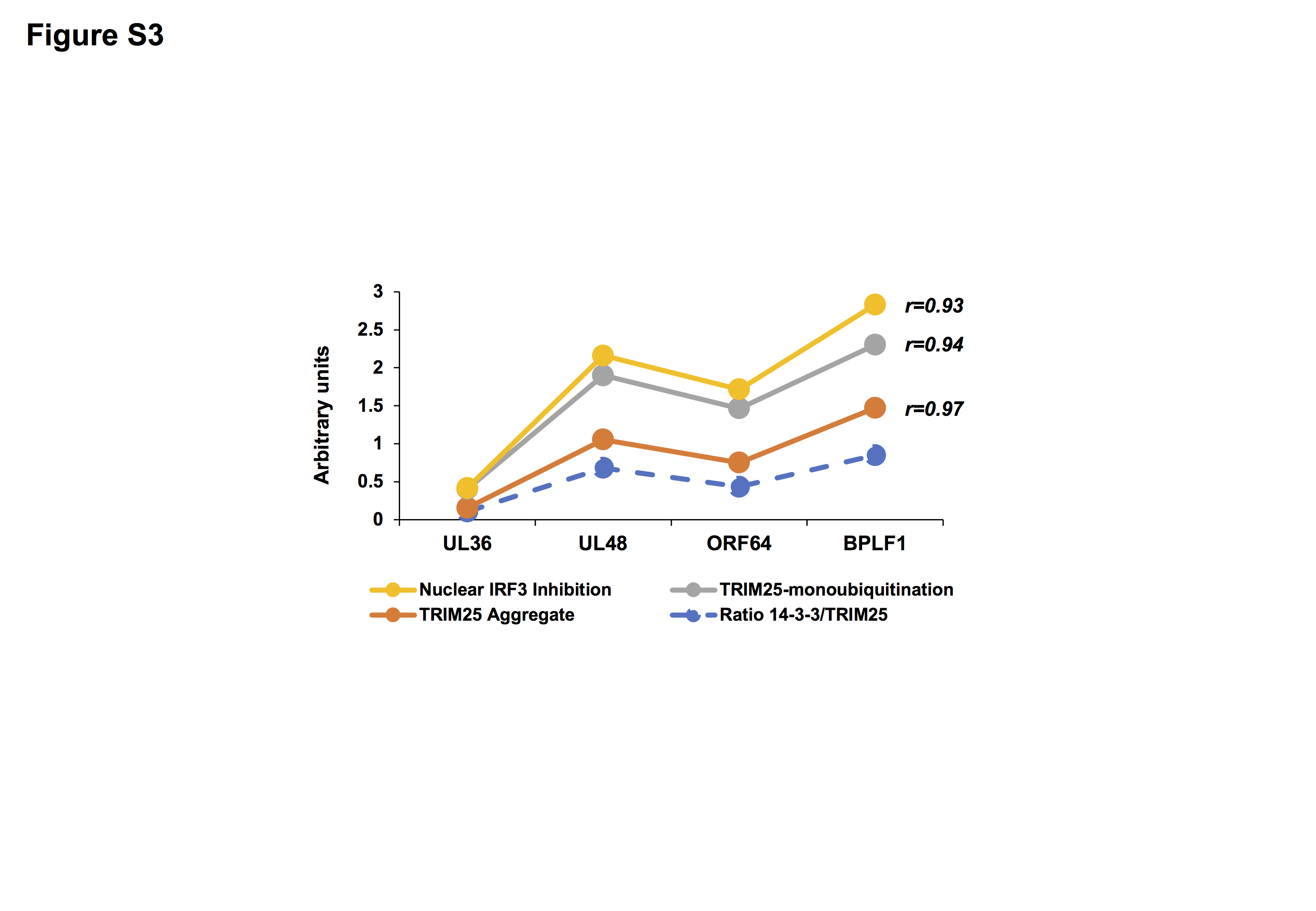

Supplement: Figure S3 — Correlation between the interaction with 14-3-3 and TRIM25 and inhibition of the IFN response. Graphic representation of the relationship between the ratio of 14-3-3/TRIM25 co-immunoprecipitation (blue dotted line) and: TRIM25 mono-ubiquitination (gray line), the formation of TRIM25 aggregates (orange line), inhibition of IRF3 nuclear translocation (yellow line). The data are expressed in arbitrary units. Higher 14-3-3/TRIM25 ratio correlates with increased TRIM25 aggregate formation (r = 0.97), TRIM25 ubiquitination (r = 0.93) and inhibition of IRF3 nuclear translocation (r = 0.94). [file Image_3.TIFF]
